# The potential interruptive effect of tinnitus-related distress on attention

**DOI:** 10.1038/s41598-020-68664-1

**Published:** 2020-07-17

**Authors:** Sook Ling Leong, Stephanie Tchen, Ian H. Robertson, Ola Alsalman, Wing Ting To, Sven Vanneste

**Affiliations:** 1 Trinity Institute of Neuroscience, Trinity College Dublin, Dublin, Ireland; 2 Lab for Clinical and Integrative Neuroscience, School of Behavioral and Brain Sciences, The University of Texas at Dallas, Texas, USA; 3 Lab for Clinical and Integrative Neuroscience, School of Psychology, Global Brain Health Institute and Institute of Neuroscience, Trinity College Dublin, College Green 2, Dublin, Ireland

**Keywords:** Risk factors, Signs and symptoms

## Abstract

The mechanism through which tinnitus affects attention is unclear. This study examines whether distress mediates the relationship(s) between tinnitus and sustained, selective and executive attentions as well as response inhibition. Eighteen participants with tinnitus and fifteen controls completed the Counting Stroop, Vigilance and Stop Signal tasks. Tinnitus distress was assessed using the Tinnitus Questionnaire (TQ), severity of depressive mood states examined using the Beck Depression Inventory-II, and general distress assessed using the Hospital Anxiety and Depression Scale. Tinnitus participants had significantly slower reactions during the Vigilance task (*F* = 4.86, *p* = .035), and incongruent trials of the Cognitive Counting task (*F* = 3.45, *p* = .045) compared to controls. Tinnitus-related distress significantly mediated the effect of tinnitus in incongruent trials (TQ: Sobel test *t* = 1.73, *p* = .042) of the Cognitive Counting Task. Complaints of distress and concentration difficulties are common amongst tinnitus patients in clinical settings and these afflictions have been shown to negatively impact an individual’s quality of life. If confirmed in future studies, results suggest that distress may be an important factor in the causal mechanism between tinnitus and attention.

## Introduction

Tinnitus, is the perception of an auditory signal without the presence of an external source^1^. Due to a lack of a standardized definition, this neurological condition has been reported to have a wide prevalence range varying between 5 and 43% worldwide^2^. A significant proportion of those experiencing tinnitus report a diverse array of comorbid symptoms including distress, depression, anxiety, sleep disturbances, and concentration difficulties^3-5^. The exact underlying mechanism of tinnitus is unknown^1^, and even though numerous treatments, extending from behavioral and sound therapies, to pharmacological treatments can alleviate symptoms and complications, there is no available cure for tinnitus^1,6^.

Neuroimaging studies of tinnitus patients have shown that, besides alterations in the central auditory pathways, there appears to be abnormalities in nonauditory brain areas^7^. It has been theorized that brain regions imperative in the control of attention, specifically the salience, cognitive control and affect networks are involved in tinnitus^7^. Keeping in mind that cognitive capacity is limited^8^, an increased spontaneous firing in the central auditory cortex could lead to increased prioritization of auditory salience and its associated emotional distress, eventuating in a reduced top-down regulation of the cognitive control network^7^. In other words, the constant engagement or failure to switch attention from the tinnitus sound because of poor cognitive and emotional controls could result in impaired attention on other tasks. Yet, recent neuropsychological tinnitus models have suggested that deficits in attention mechanisms should not only be considered a symptom of tinnitus but a contributing factor to the maintenance of tinnitus itself^9^. It could be postulated that shortcomings of the top-down cognitive inhibitory process together with changes in the auditory system facilitate the ability of this phantom sound to reach consciousness, and its persistence over time^9,10^.

Attention is not a unitary phenomenon but has different subtypes^11^. One established model is the three-component model of Posner and Petersen, 1990^12^ suggesting three attentional sub-components: (1) orienting or sustained attention (i.e. achieving and maintaining an alert state), (2) selective attention (i.e. selecting task-relevant stimuli and ignoring task-irrelevant stimuli) and (3) executive attention (i.e. resolving conflict among responses). Research on attention deficits in tinnitus are scare with obscurity on whether tinnitus depletes attentional resources in general^11,13-16^, manifesting in poorer performance on all conditions of attentional task or that tinnitus negatively impacts the different attentional sub-components, namely sustained attention^14^, selective attention^16^, or executive attention^13,17^.

Dysfunctional attention mechanisms in tinnitus are usually reflected in significantly longer response times on attention tasks compared to controls^13-19^. The classic color Stroop task^20^ is a complex attention-demanding cognitive task that measures attentional interference and attentional control by assessing the reaction time to name the color of a word that is incongruent with the meaning of the word (e.g. word ‘blue’ in red ink, answer is red). Among healthy controls, the latency to color-name incongruent words is typically increased when compared to congruent words (i.e. words that matched the color e.g. word ‘blue’ in blue ink)^21^.

The Stroop task in tinnitus research has been used as a measure of selective and executive attentions with mixed outcomes^11^. Initial studies have reported that tinnitus participants were overall slower in both congruent and incongruent trials compared to controls, indicating a dysfunction in selective attention^13,16,17^. A later study however, demonstrated that in comparison to controls, tinnitus participants were slower only in the incongruent trials, proposing a deficit in executive attention^19^. Yet, when examined exclusively in tinnitus participants without hearing loss, there were no significant differences between the tinnitus and control groups in Stroop performances^22^, suggesting hearing loss as a potential confounder. A meticulous assessment of these studies revealed that heterogeneity in age, gender, cognitive ability, hearing loss, depression and anxiety could potentially contribute to inconsistent results^11^. As for sustained attention, there is currently a lack of compelling evidence supporting the proposition that tinnitus negatively affects sustained attention^11,14^.

Another avenue for understanding the relationship between attention and tinnitus is to investigate the effect of tinnitus-related distress on attention. Given the interconnectedness of the salience, cognitive control and affective networks in tinnitus, the degree of impairment in attention could be a consequence of not only tinnitus itself but its associated disorders such as distress, depression, or anxiety. Research has shown, for instance, that deficits in cognitive processing are more apparent in highly distressed tinnitus patients.^15-17,19^.

Therefore, in the present study, we investigated whether distress mediates the relationship(s) between tinnitus and sustained, selective, and executive attentions as well as response inhibition, the latter of which served as a control, i.e. to determine whether the mediating effect is limited to attention or applies to cognition more broadly. Furthermore, to avoid the pitfalls encountered by previous studies, we controlled for potentially confounding factors including age, gender, hearing loss, cognitive ability, depression, anxiety, and sleep quality. Finally, we examined correlations between tinnitus-related distress and performance on our attention tasks, which we describe in detail in the “Methods” section.

## Methods

### Participants

**Figure 1:**
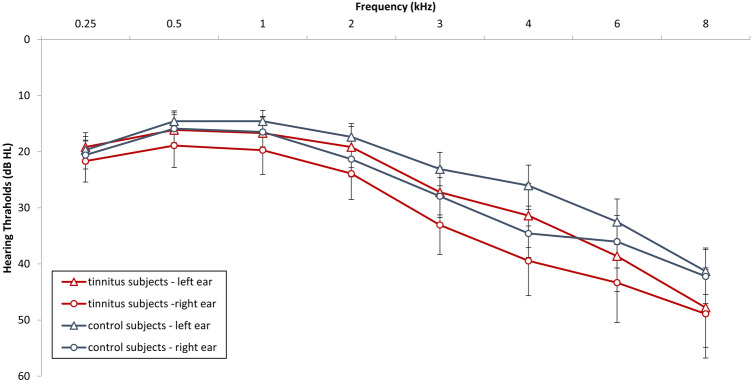
Auditory thresholds for the left and right ear for control subjects and tinnitus subjects. Both tinnitus and control groups had similar auditory thresholds when individual frequencies were investigated.

Eighteen participants with tinnitus and 15 controls matched for age, gender, and hearing loss (see Fig. 1) were recruited through flyers posted at the University of Texas at Dallas and the Callier Center for Communication Disorders as well as by word of mouth. All participants gave their informed consent. This study was performed in accordance with the Helsinki declaration standards and the study protocol was approved by the Institutional Review Boards of the University of Texas at Dallas (IRB protocol #15-30). Participants displayed, at worst, rudimentary reading capabilities and absence of neurological/cognitive health-related abnormalities. Tinnitus participants had to confirm the occurrence of tinnitus for at least six months to meet our inclusion criterion. Individuals with pulsatile tinnitus, Ménière’s disease, otosclerosis, chronic headache, neurological disorders such as brain tumors, traumatic brain injury or stroke and individuals being treated for mental disorders were not included in the study. This was assessed by a neurological examination. Participants needed to be able to understand and read the informed consent and instructions to be able to be enrolled in this study. All participants provided a general otologic history and undertook a hearing evaluation that included air-conduction, pure-tone audiometry at 250–8,000 Hz. Audiological tinnitus evaluations comprised pitch and loudness matching to ascertain the characteristic frequency and intensity of each participant’s tinnitus. Participants were not compensated for partaking in the study. The number of participants was determined based on previous research.

### Self-reported questionnaires

For all participants, the Beck Depression Inventory-II (BDI-II)^23^ was used to evaluate the severity of depressive mood states; the Hospital Anxiety and Depression Scale (HADS)^24^ to examine general distress; the Pittsburgh Sleep Quality Index (PSQI)^25^ to assess quality of sleep; and the Montreal Cognitive Assessment (MoCA)^26^ to provide a global understanding of participants’ cognitive abilities. In addition, tinnitus participants completed the Tinnitus Questionnaire (TQ)^27^ to assess tinnitus-related distress and the Visual Analog Scale (VAS)^28^ for evaluation of tinnitus loudness.

### Behavioral tasks

#### Counting Stroop task (selective and executive attention)

The task requires subjects to count the number of words in a display of words that denote a number (e.g. the word ‘three’ written twice) by pushing one of four buttons^29,30^. Reaction time to count these words is typically greater in the incongruent condition (e.g. the word ‘three’ written twice) compared to the congruent (e.g. the word ‘two’ written twice), i.e. the Stroop effect. Cognitive words represented written numbers (i.e. one, two, three, four), similar to the study of Bush and colleagues (1998)^29^. The experiment consisted of four blocks with a break after the second block. Block 1 and 2 each consisted of 40 trials (12 congruent trials, 12 incongruent trials, randomly alternated). These two blocks were repeated after a one-minute break where participants could take a break and relax their fingers. Each trial was displayed for 1.5 s followed by a fixation cross for 0.5 s (inter stimulus interval = 0.5 s). Prior to the four test blocks, the subjects first completed one practice block with five novel neutral words (i.e. porch, corridor, dishwasher, fan, mailbox) presented twice (i.e. 10 words) with feedback to make sure they practiced well enough on the task components of counting and button pressing, but without seeing the words to be tested in the test blocks. The experiment was displayed on a computer screen using Presentation software (Version 18.0, Neurobehavioral Systems, Inc., Berkeley, CA, www.neurobs.com), and participants reported their answers by pushing one of four buttons on the keyboard using their right or left index or middle finger (1 = middle finger left hand, 2 = index finger left hand 3 = index finger right hand, 4 = middle finger right hand) (Fig. 1A).

#### Vigilance task (sustained attention)

Each participant completed 720 vigilance trials in two blocks. Participants were instructed to focus on a fixation cross in the middle of a white screen. For each trial, a square appeared randomly for 1.4 s and participants were instructed to click the mouse only when they observed the square at the top of the screen^31^ (Fig. 1B).

#### The Stop Signal task (response inhibition)

**Figure 2:**
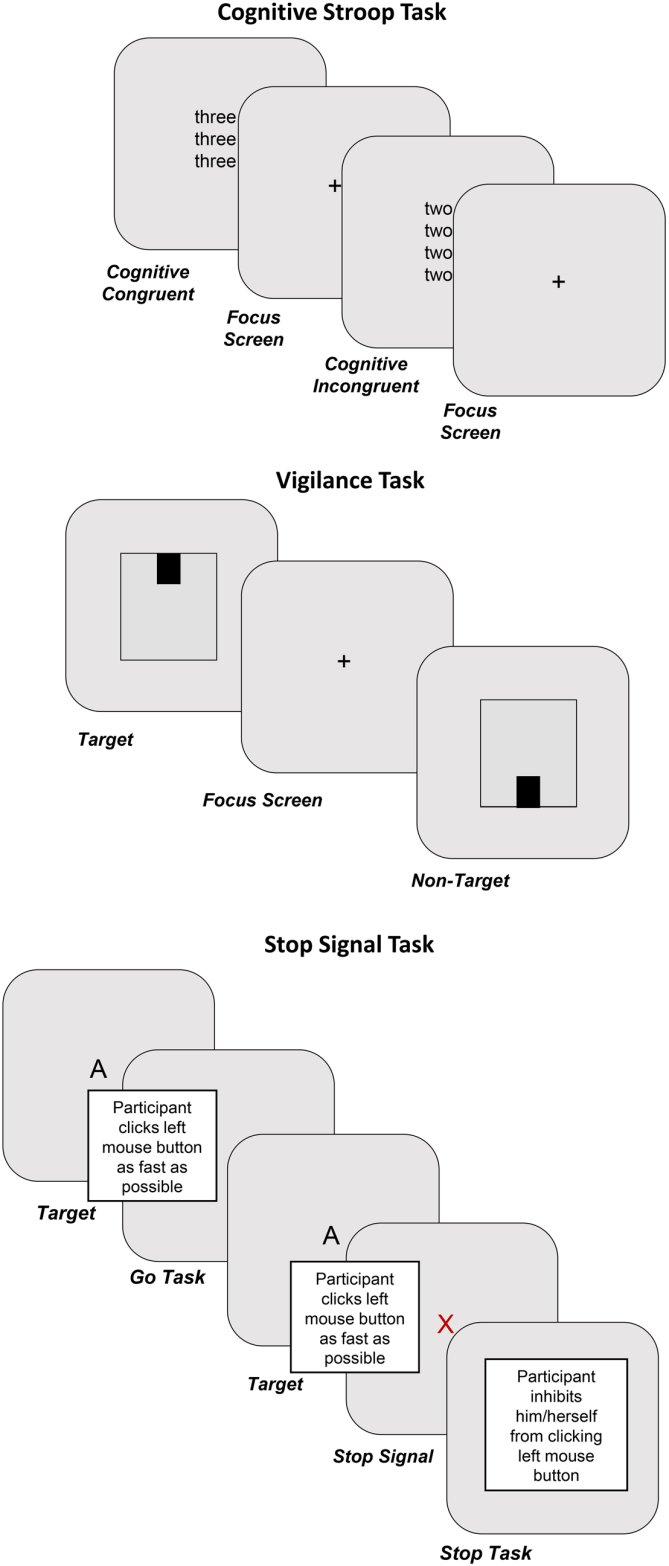
Schematic overview of the different tasks. (**A**) Cognitive counting Stroop task, (**B**) Vigilance task, and (**C**) Stop-Signal tasks.

The Stop Signal task evaluated the cognitive control process of response inhibition resulting from changes in environmental demands, independent from the attention network^32^. This task was included as a control to determine whether tinnitus affects only the attention network, or whether it also affects cognitive processes (in this case, response inhibition). During this task, participants were presented with either a flash of “A” or “Z” on the screen. They were instructed to click the left button as quickly as possible when “A” flashes and the right button as quickly as possible when “Z” flashes. However, when a red “X” was presented immediately following either letter, participants were asked to refrain from clicking. Each participant completed 200 randomized stop-signal trials, where the letters “A” or “Z” were randomly presented for 50–500 ms (Fig. 2C).

### Statistical analyses

Descriptive statistics, independent t-tests, and Pearson’s Chi Square tests were conducted to examine differences in self-reported measures between tinnitus and control participants. Repeated measures ANOVAs were utilized to investigate interaction effects of condition and group for the Stroop and Vigilance tasks. Pearson’s correlation was conducted to examine relationships between self-reported measures and computerized tasks. In addition, a mediation analysis was utilized to examine whether distress mediated the relationship between tinnitus loudness and the attention tasks in tinnitus participants (cognitive counting Stroop task, and Vigilance task). Where *p* < 0.05, results were deemed significant. All analyses were conducted using SPSS Statistics for Windows, version 32 (IBM Corp., Armonk, N.Y., USA).

## Results

### Descriptive statistics and controlled factors

Independent t-tests showed no significant differences for MoCA, HADS, PSQI and BDI-II between tinnitus and control participants (Table 1). Pearson’s Chi Square tests revealed no significant difference in gender between groups, while multivariate analysis demonstrated that the two groups did not differ in mean hearing loss over all frequencies (Table 1). Both groups had similar auditory thresholds when individual frequencies were investigated (Fig. 2).

**Table 1:** Descriptive statistics and controlled factors.

	Control *M,* (*Sd*)	Tinnitus *M,* (*Sd*)	Significance
Age (years)	51.19 (14.66)	49.44 (18.02)	(*F* = .094, *p* = .76)
Gender (male/female)	10/6	11/7	(*χ*^2^ = .93, *p* = .61)
Mean hearing loss (dB HL)	22.42 (9.89)	28.77 (15.58)	(*F* = 2.43, *p* = .18)
MoCA	29.31 (1.14)	29.17 (1.15)	(*F* = .13, *p* = .71)
PSQI	4.38 (2.451)	5.67 (3.61)	(*F* = 1.45, *p* = .23)
BDI	4.06 (2.69)	4.78 (4.96)	(*F* = .26, *p* = .61)
Depression (HADS)	2.18 (2.04)	2.78 (1.93)	(*F* = .75, *p* = .39)
Anxiety (HADS)	1.81 (1.87)	1.83 (1.72)	(*F* = .001, *p* = .97)
TQ	–	14.39 (11.27)	–
Loudness (VAS)	–	3.69 (2.50)	–

In tinnitus participants, significant correlations were obtained between TQ with PSQI (r = 0.59, *p* = 0.005) and BDI-II (r = 0.69, *p* = 0.001), but not with MoCA (r = − 0.19, *p* = 0.23), HADS depression (r = 0.30, *p* = 0.22) or HADS anxiety (r = − 0.13, *p* = 0.64). For tinnitus loudness, significant positive correlations were demonstrated with TQ (r = 0.73, *p* = 0.001), but not with MoCA (r = − 0.09, *p* = 0.73), HADS depression (r = 0.03, *p* = 0.89), HADS anxiety (r = − 0.03, *p* = 0.91), PSQI (r = 0.24, *p* = 0.34) or BDI-II (r = 0.36, *p* = 0.15) (Table 1).

### Computerized tasks

#### Cognitive counting Stroop task

**Figure 3:**
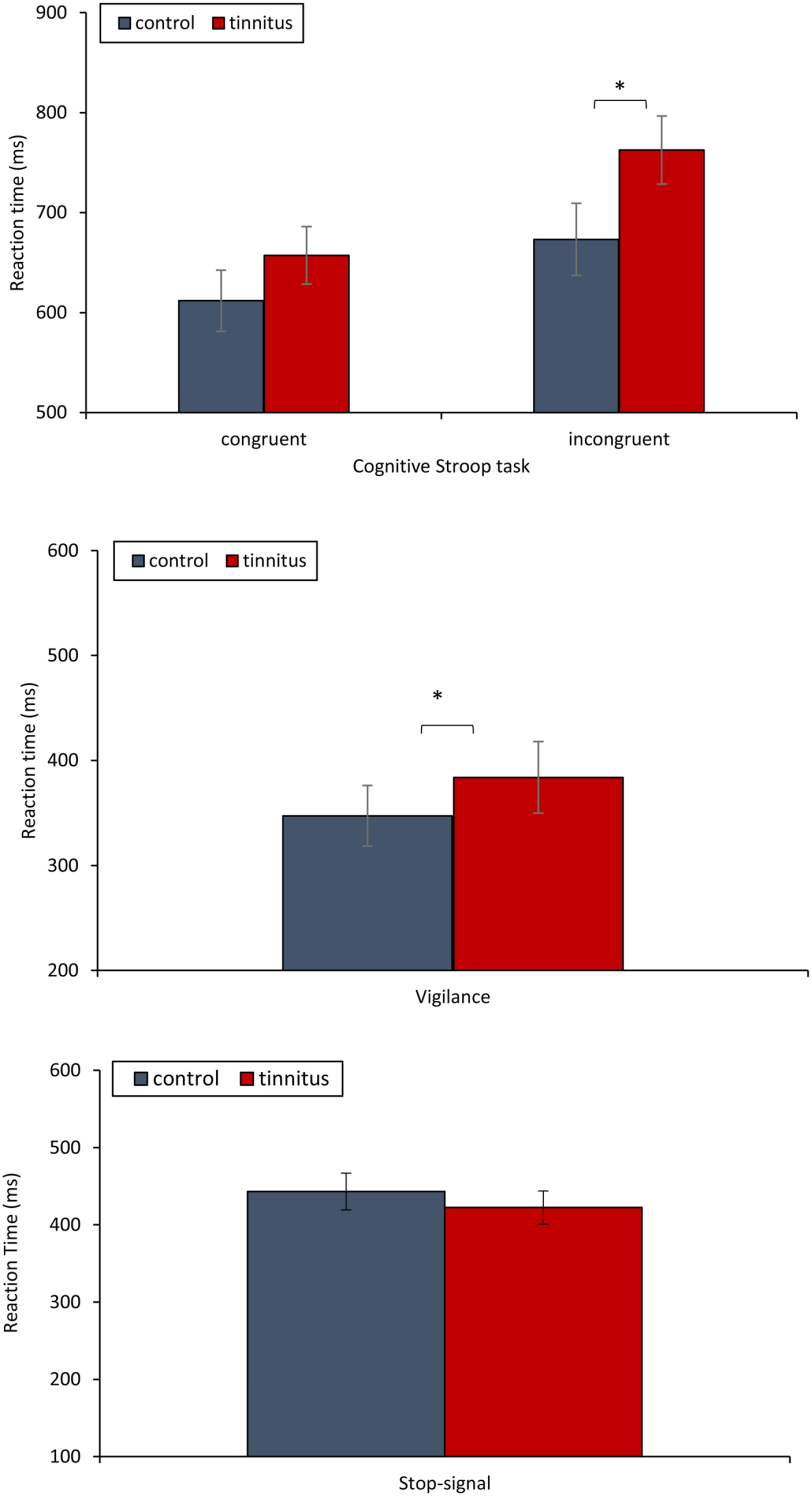
Comparison between control and tinnitus participants in reaction times for the different tasks. (**A**) Cognitive counting Stroop task, (**B**) Vigilance task, and (**C**) Stop-Signal task.

Repeated measures ANOVA revealed a significant main effect (*F* = 96.78, *p* < 0.001) for condition (congruent versus incongruent), but not for group (tinnitus versus control) (*F* = 2.22, *p* = 0.15). Both groups were significantly slower for incongruent (*M* = 720.60 ms, *SD* = 149.24 ms) compared to congruent trials (*M* = 635.88 ms, *SD* = 122.71 ms). There was a significant interaction effect between condition and group (*F* = 6.79, *p* = 0.014). Tinnitus participants (*M* = 657.20 ms, *SD* = 117.69 ms) did not significantly (*F* = 1.16, *p* = 0.29) differ for congruent trials in comparison to controls (*M* = 611.90 ms, *SD* = 127.57 ms). A simple contrast analysis for the incongruent trial showed a significant effect (*F* = 3.45, *p* = 0.045), demonstrating that tinnitus participants (*M* = 762.72 ms, *SD* = 148.22 ms) were slower in processing incongruent trials than controls (*M* = 673.22 ms, *SD* = 139.92 ms). Noteworthily, the interaction effect (*F* = 4.64, *p* = 0.041) remained after controlling for MoCA, PSQI, hearing loss, age, HADS, and BDI. See Fig. 3A for a summary of Cognitive Stroop task results.

#### Vigilance task

One-way ANOVA showed a significant effect (*F* = 4.86, *p* = 0.035), demonstrating that tinnitus participants (*M* = 384.04 ms, *SD* = 56.79 ms) were slower on the Vigilance task in comparison to controls (*M* = 347.39 ms, *SD* = 36.57 ms). After controlling for MoCA, PSQI, hearing loss, age, HADS, and BDI, the effect remained (Fig. 3B).

#### Stop-signal task

A one-way ANOVA showed no significant effect (*F* = 1.32, *p* = 0.26) between the tinnitus group and controls for the Stop-Signal task (Fig. 3C).

### Correlation and mediation analyses

**Figure 4:**
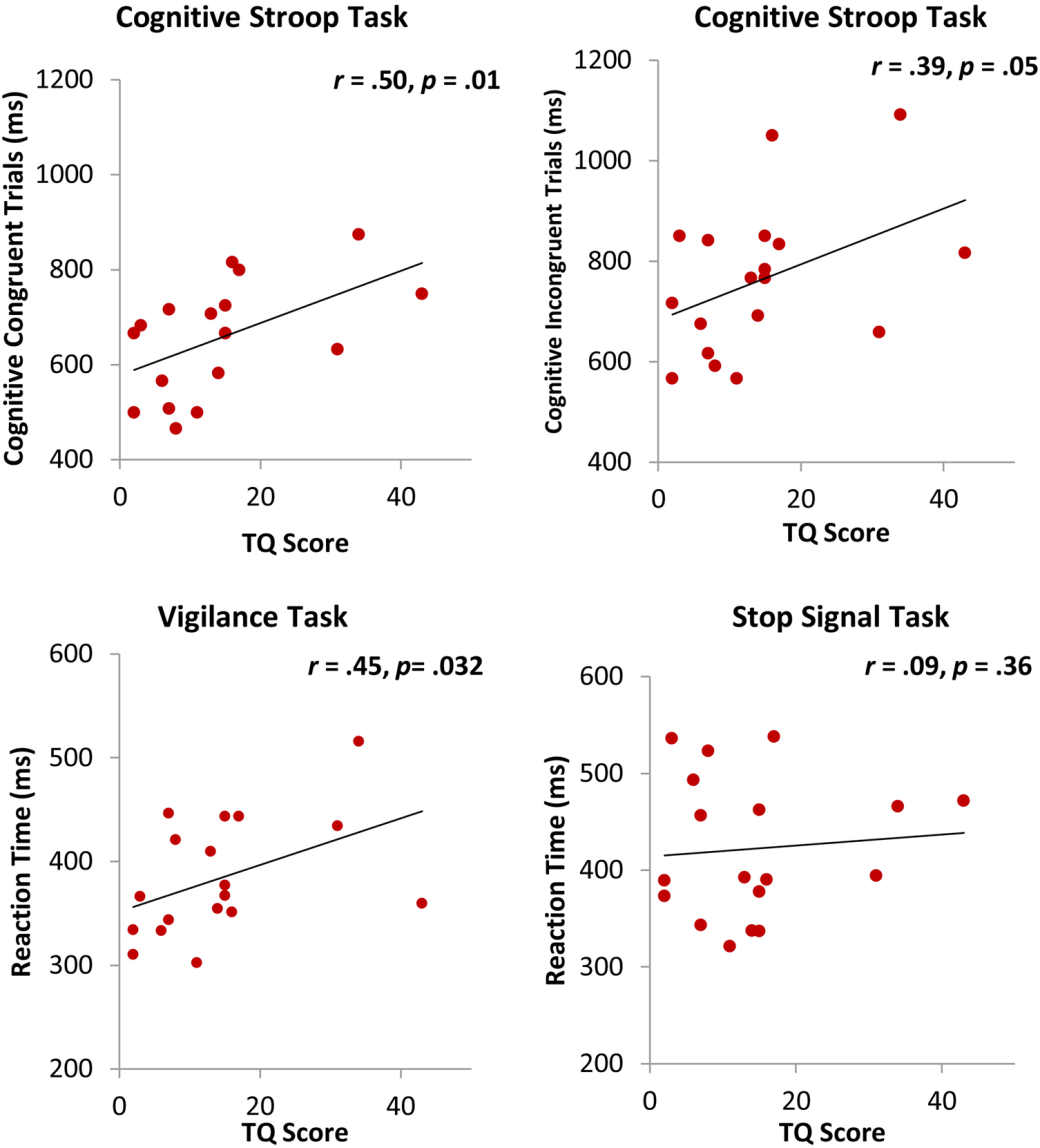
Correlation analysis between TQ scores and reaction times for the different tasks. (**A**) Congruent trials of the cognitive counting Stroop task, (**B**) Incongruent trials of the Cognitive counting Stroop task, (**C**) Vigilance task, and (**D**) Stop-Signal task.

**Figure 5:**
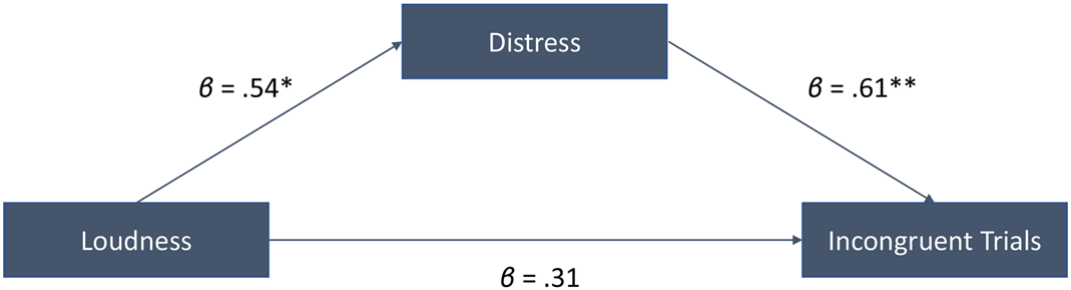
Mediation analysis between loudness, distress, and incongruent trials controlling for age, gender, hearing loss, cognitive ability, depression, anxiety, and sleep quality.

Pearson’s correlations revealed significant positive correlations for TQ in the cognitive congruent (r = 0.50, *p* = 0.01) (Fig. 4A) but not the incongruent trials (r = 0.61, *p* = 0.007) (Fig. 4B). There was a significant correlation for the TQ with the Vigilance tasks (r = 0.45, *p* = 0.03) (Fig. 4C). Results remained significant after controlling for HADS, BDI, hearing loss, age, VAS loudness, PSQ, and MoCA using partial correlation. (Fig. 4). We assessed whether tinnitus-related distress mediated the association between tinnitus loudness and the Counting Stroop task. We found that tinnitus-related distress significantly mediated the effect of tinnitus in incongruent trials (TQ: Sobel test *t* = 1.97, *p* = 0.042; see Fig. 5). There was a significant positive correlation between BDI and Vigilance task (r = 0.43, *p* = 0.04) but not after controlling for TQ (r = 0.19, *p* = 0.23). There were no significant correlations between BDI and cognitive congruent or incongruent trials.

## Discussion

Previous research examining the associations between tinnitus and attention have produced mixed results^9,13,19^. Moreover, a published review^11^ of this topic proposed that future experimental studies manipulating the effect of tinnitus on attention should not only include an assessment of all three sub-components of attention but include identified potential confounders, namely age, gender, cognitive ability, hearing loss, depression and anxiety. The question on which exact attention networks or sub-components of attention are altered by tinnitus is important for the development of targeted treatments. For example, establishment of the exact attention deficit could dictate which aspect of attention should be targeted during cognitive training. Also, the identification of the dysfunction attention network in tinnitus means more tailored and precise targets for neuromodulation.

Results from this study revealed decrements in certain attentional domains among tinnitus participants compared to controls. In contrast to previous studies^14,33^, the tinnitus group had significantly poorer sustained attention compared to controls, reflected by their slower reaction time during the Vigilance task. Although a Stroop effect was observed in both the tinnitus and control groups, consistent with previous studies^19^, tinnitus participants demonstrated significantly slower reaction times for incongruent trials compared to controls in the cognitive counting Stroop task. Our results suggest that attention deficits in tinnitus may not be a consequence of a general depletion of attentional resources^13-16^, as both groups did not differ in response time in the congruent trials of the cognitive counting Stroop task. Our results further indicate that a general decrease in ability to inhibit their impulses is an unlikely explanation, given the non-significant results of the Stop-Signal task. The significant positive correlations between reaction times for all performed attentional tasks with tinnitus-related distress but not with tinnitus loudness imply that tinnitus-related distress plays a more important role in impairing attention. Importantly, if these results from the mediation model are repeatable in future studies, they suggest that distress may be a key factor in the underlying causal mechanism between tinnitus and attention.

Theoretically, the premise of a dysfunctional top-down attention system provides a unifying framework for the interpretation of current results^9,34^. Non-auditory brain areas that have been consistently shown to be involved in tinnitus include the middle temporal and para-hippocampus, the precuneous and the dorsal attention network^7,35^. These brain regions, which are functionally connected to the auditory cortex,^36^ are involved in regulating the focus of awareness and are important components of the top-down, executive control of attention^37^. Accordingly, in tinnitus, an impaired network would result in a reduced ability to resolve conflict among responses (i.e. executive attention) and inhibit the voluntary division of attentional resources^37^. Moreover, a recent resting-state functional magnetic resonance imaging study reported that tinnitus distress is associated with modifications of functional connectivity within regions of the executive network as well as the salience and default mode networks, suggesting that tinnitus distress is inevitably linked to attention of the tinnitus sound^35^. Thus, these underlying mechanisms, if exploited clinically should focus on actively redirecting attention away from the tinnitus sound as well as the related distress.

In a clinical setting, complaints of distress are common amongst tinnitus patients^38,39^. Yet, treatment strategies focusing on managing tinnitus-related distress are limited^40,41^. Several pharmacological treatments influencing tinnitus distress are available; however, a significant proportion of patients are resistant to these agents^40^. One treatment strategy that has been shown to be effective in redirecting attention away from tinnitus and its associated distress is cognitive behavior therapy (CBT)^42^. Theoretically, the cognitive model of CBT is a well-established treatment approach, relying on the brain’s top-down mechanism. In tinnitus, CBT interventions consider that attention to the tinnitus sound as a key causal factor in the maintenance and amplification of distress^42,43^. Therefore, tinnitus CBT treatment approaches facilitate changes in attentional focus through the development of adaptive coping thoughts^42^. Ultimately, even though the auditory perception is not eliminated, there is a reduction in negative response to tinnitus^42^.

Another non-pharmacological avenue that could be explored is non-invasive neuromodulation. Studies using transcranial magnetic stimulation and bifrontal transcranial direct current stimulation to modulate the dorsal lateral prefrontal (DLPFC) and anterior cingulate cortex (ACC) have shown to successfully divert attention from tinnitus sound and the associated distress^44,45^. The dorsolateral prefrontal cortex is suggested to be involved in auditory attention, resulting in top-down modulation of auditory processing and emotion regulation, while the ACC is involved in part of a distress network that plays a role in the salience of tinnitus^44,45^.

Although promising, treatment approaches aimed at rehabilitating the top-down execution attention network are based on postulated theoretical frameworks from tinnitus and attention research. To further pursue executive function rehabilitation as an effective treatment for tinnitus, functional neuroimaging studies are needed to establish the relationship between dysfunctional top-down executive control and alterations of the sub-categories of attention.

In conclusion, the present study showed that sustained and executive attention processes were affected in tinnitus compared to control participants after controlling for previously identified confounding factors. In addition, further analyses unveiled that the relationship between tinnitus and attention was mediated by distress. We posit that deficiencies in the top-down executive control network negatively impact performance in the various attentional tasks. The hyper-attention towards this distressful phantom sound can be alleviated through therapies that alter attention from the auditory percept. Furthering our understanding regarding the neurobiological underpinnings of the imbalanced interactions between the cognitive control, salience, and affective networks in tinnitus is an important step towards elucidating the role of attention in tinnitus, and subsequently the development of more tailored treatment strategies.
